# Efficacy of chlorfenapyr-pyrethroid and piperonyl butoxide-pyrethroid long-lasting insecticidal nets (LLINs) compared to pyrethroid-only LLINs for malaria control in Côte d’Ivoire: a three group, cluster randomised trial

**DOI:** 10.1186/s13063-024-07969-2

**Published:** 2024-02-28

**Authors:** Colette Sih, Natacha Protopopoff, Alphonsine A. Koffi, Ludovic P. Ahoua Alou, Edouard Dangbenon, Louisa A. Messenger, Manisha A. Kulkarni, Marius G. Zoh, Soromane Camara, Serge B. Assi, Raphael N’Guessan, Jackie Cook

**Affiliations:** 1 Faculty of Epidemiology and Population Health, Department of Infectious Disease Epidemiology, London School of Hygiene and Tropical Medicine, London, WC1E 7HT UK; 2 Faculty of Infectious and Tropical Diseases, Disease Control Department, London School of Hygiene and Tropical Medicine, London, WC1E 7HT UK; 3 Institut Pierre Richet (IPR)/Institut National de Santé Publique (INSP), Bouaké, Côte d’Ivoire; 4 Department of Environmental and Occupational Health, School of Public Health, University of Nevada, Las Vegas, NV 89154 USA; 5 School of Epidemiology & Public Health, Faculty of Medicine, University of Ottawa, Ottawa, ON Canada; 6 Medical Research Council (MRC) International Statistics and Epidemiology Group, London School of Hygiene and Tropical Medicine, London, WC1E 7HT UK

**Keywords:** Cluster randomised trials, Côte d’Ivoire, Dual-active long-lasting insecticidal nets, Entomological inoculation rate, Insecticides, Insecticide resistance, Interceptor® G2, MAGNet®, Malaria case incidence, Malaria prevalence, VEERALIN®

## Abstract

**Background:**

The massive scale-up of long-lasting insecticidal nets (LLIN) has led to a major reduction in malaria burden in many sub-Saharan African (SSA) countries. The World Health Organization (WHO) has recently issued a strong recommendation for the use of chlorfenapyr-pyrethroid LLINs compared to standard pyrethroid-only LLINs in areas of high insecticide resistance intensity. However, there is still a lack of conclusive evidence on the efficacy of piperonyl butoxide-pyrethroid (PBO-py) LLINs, especially in West Africa, where vector composition and resistance mechanisms may be different from vectors in East Africa.

**Methods:**

This is a three-arm, superiority, triple-blinded, cluster randomised trial, with village as the unit of randomisation. This study conducted in Côte d’Ivoire will evaluate the efficacy on epidemiological and entomological outcomes of (1) the control arm: MAGNet® LN, which contains the pyrethroid, alpha-cypermethrin, (2) VEERALIN® LN, a net combining the synergist PBO and alpha-cypermethrin, and (3) Interceptor® G2 LN, which incorporates chlorfenapyr and alpha-cypermethrin, two adulticides with different mechanisms of action. A total of 33 villages with an average of 200 households per village will be identified, mapped, and randomised in a ratio of 1:1:1. Nets will be distributed at a central point following national guidelines with 1 net for every 2 people. The primary outcome of the trial will be incidence of malaria cases (confirmed by rapid diagnostic test (RDT)) in a cohort of 50 children aged 6 months to 10 years in each cluster, followed for 12 months (active case detection). Secondary outcomes are cross-sectional community prevalence of malaria infection (confirmed by RDT) in the study population at 6 and 12 months post-intervention (50 randomly selected persons per cluster), vector density, entomological inoculation rate (EIR), and phenotypic and genotypic insecticide resistance at baseline and 12 months post-intervention in 3 sentinel villages in each treatment arm.

**Discussion:**

In addition to generating further evidence for next-generation LLINs, this study will also provide the first evidence for pyrethroid-PBO nets in a West African setting. This could further inform WHO recommendations on the pragmatic use of pyrethroid-PBO nets.

**Trial registration:**

ClinicalTrials.gov NCT05796193. Registered on April 3, 2023.

## Administrative information

Note: the numbers in curly brackets in this protocol refer to SPIRIT checklist item numbers. The order of the items has been modified to group similar items (see http://www.equator-network.org/reporting-guidelines/spirit-2013-statement-defining-standard-protocol-items-for-clinical-trials/).

**Table:** 

Title {1}	Efficacy of chlorfenapyr-pyrethroid and piperonyl butoxide-pyrethroid long-lasting insecticidal nets (LLINs) compared to pyrethroid-only LLINs for malaria control in Côte d’Ivoire: a three group, cluster randomised trial.
Trial registration {2a and 2b}.	ClinicalTrials.gov, NCT05796193, registered on April 3, 2023 https://clinicaltrials.gov/ct2/show/NCT05796193?recrs=ab&cond=malaria&draw=2&rank=6
Protocol version {3}	version 1.1 of December 08, 2022
Funding {4}	This trial is funded by the Global Fund.
Author details {5a}	Colette Sih: Faculty of Epidemiology and Population Health, Department of Infectious Disease Epidemiology, London School of Hygiene and Tropical Medicine, WC1E 7HT, London, UK. Email: Colette.Sih@lshtm.ac.uk Natacha Protopopoff: Faculty of Infectious and Tropical Diseases, Disease Control Department, London School of Hygiene and Tropical Medicine, WC1E 7HT, London, UK Email: Natacha.Protopopoff@lshtm.ac.uk Alphonsine A. Koffi: Institut Pierre Richet (IPR))/Institut National de Santé Publique (INSP), Bouaké, Côte d'Ivoire. Email: koffi_alphonsine@yahoo.fr Ludovic P. Ahoua Alou: Institut Pierre Richet (IPR))/Institut National de Santé Publique (INSP), Bouaké, Côte d'Ivoire. Email: ludovicalou@gmail.com Edouard Dangbenon: Institut Pierre Richet (IPR))/Institut National de Santé Publique (INSP), Bouaké, Côte d'Ivoire. Email: dangbenonedouard@gmail.com Louisa A. Messenger: Faculty of Infectious and Tropical Diseases, Disease Control Department, London School of Hygiene and Tropical Medicine, WC1E 7HT, London, UK and Department of Environmental and Occupational Health, School of Public Health, University of Nevada, Las Vegas, NV, 89,154, USA Email: Louisa.Messenger@unlv.edu Manisha A. Kulkarni: School of Epidemiology & Public Health, Faculty of Medicine, University of Ottawa, Ottawa, Ontario, Canada. Email: Manisha.Kulkarni@uOttawa.ca Marius G. Zoh: Institut Pierre Richet (IPR))/Institut National de Santé Publique (INSP), Bouaké, Côte d'Ivoire. Email: marius_zoh@yahoo.com Soromane Camara: Institut Pierre Richet (IPR)/Institut National de Santé Publique (INSP), Bouaké, Côte d'Ivoire. Email: soromanec@gmail.com Serge B. Assi: Institut Pierre Richet (IPR))/Institut National de Santé Publique (INSP), Bouaké, Côte d'Ivoire. Email: assisergi@yahoo.fr Raphael N’Guessan: Faculty of Infectious and Tropical Diseases, Disease Control Department, London School of Hygiene and Tropical Medicine, WC1E 7HT, London, UK and Institut Pierre Richet (IPR))/Institut National de Santé Publique (INSP), Bouaké, Côte d'Ivoire. Email: Raphael.N’Guessan@lshtm.ac.uk Jackie Cook: Medical Research Council (MRC) International Statistics and Epidemiology Group, London School of Hygiene and Tropical Medicine, WC1E 7HT, London, UK and Faculty of Epidemiology and Population Health, Department of Infectious Disease Epidemiology, London School of Hygiene and Tropical Medicine, WC1E 7HT, London, UK. Email: Jackie.Cook@lshtm.ac.uk
Name and contact information for the trial sponsor {5b}	Trial Sponsor: London School of Hygiene and Tropical Medicine Sponsor’s Reference: 2022-KEP-898 Contact name: Naomi Panteli Address: London School of Hygiene & Tropical Medicine, Keppel Street, London WC1E 7HT Telephone: + 44 (0) 207 927 2102 Email: RGIO@lshtm.ac.uk
Role of sponsor {5c}	The Global Fund has no role in the study design, data collection, management, analysis, interpretation, or the decision to submit the report for publication. The Principal Investigator (PI), Jackie Cook and Co-PI Raphael N’Guessan, will have full access to all the data in the study and will have final responsibility for the decision to submit for publication.

## Introduction

### Background and rationale {6a}

Malaria is a life-threatening disease caused by parasites of the *Plasmodium species* and transmitted through the bite of an infected female *Anopheles* mosquito [1]. It was estimated that half of the world’s population was at risk for malaria in 2021 [1], with 247 million cases recorded worldwide that year [2]. In areas of moderate to high malaria transmission, long-lasting insecticidal treated bed nets (LLIN) have been the major driver of declines in the transmission and burden of malaria [2, 3]. The increasing spread of resistance to pyrethroids has heightened the need for new insecticides to maintain gains made in malaria control in the past 20 years [4]. Recently, a suite of new insecticides have become available to treat mosquito nets [5].

The first dual active ingredient (AI) LLIN (Olyset™ Plus) combined a pyrethroid (py) with the synergist piperonyl butoxide (PBO), which inhibits P450 oxidases responsible for pyrethroid resistance. Piperonyl butoxide-pyrethroid mixture (PBO-py) LLINs have been available for some years but have not been widely used for lack of conclusive evidence favouring them over standard pyrethroid LLINs (py-LLINs). Several cluster randomised controlled trials (cRCTs) have now shown the superior efficacy of these nets [6, 7] and since the World Health Organization (WHO) recognised the public health value of bi-treated PBO-py LLINs, they were provided an interim endorsement as a new class of vector control products [8]. However, while PBO-Py-LLINs have been scaled up in many sub-Saharan African (SSA) countries following the WHO recommendation [8], there is currently no epidemiological evidence that these nets are more effective than py-LLINs in West Africa [9]. Only a small village cluster randomised controlled trial (cRCT) comparing PBO-py LLIN vs py-LLIN on entomological outcomes was conducted in Mali but did not report a significant reduction in indoor vector resting density or parity rate using PBO-py LLINs [10]. The trial was, however, insufficiently powered and arms were not balanced at baseline with higher mosquito densities in PBO-py LLIN villages.

Our trial will fill this gap and provide the first assessment of PBO-py LLIN efficacy in West Africa. It will also provide first evaluation of VEERALIN®LN in a cRCT, a brand of PBO-py LLIN that has demonstrated higher killing effect compared to py-LLINs in experimental hut trials in Côte d’Ivoire [11]. The public health recommendation of PBO-py LLIN encompasses all the PBO-py LLIN brands and a total of 7 have been WHO pre-qualified based on superior efficacy in experimental hut trials with entomological outcomes. However, brands may differ in terms of their chemical and physical specifications, pyrethroid treatment (permethrin, alpha-cypermethrin or deltamethrin) and concentration, and PBO concentration and location on the net (all net panels or only the roof). An additional trial with a different PBO-py LLIN brand and chemistry in West Africa could be used to refine the WHO recommendations and answer outstanding questions regarding this type of nets.

The other intervention product that is being evaluated in the cRCT is Interceptor® G2 LN, which is coated with a mixture of the pyrrole insecticide, chlorfenapyr, and the pyrethroid, alpha-cypermethrin. Interceptor® G2 LN has demonstrated superior efficacy compared to py-LLIN in Tanzania [12] and Benin [13] on malaria prevalence and incidence over 2 years. Based on those results, WHO has strongly recommended chlorfenapyr-py LLIN (CFP-py) as a new public health intervention to control pyrethroid-resistant vectors in SSA [14].

Similarly to the rest of SSA, Côte d’Ivoire, has seen malaria cases rise in the past 10 years despite moderate usage (59%) of standard py-LLINs by children < 5 years old reported in the last Demographic Health Survey (DHS) conducted in 2021 [15]. The country is among the top ten with the highest rates of malaria cases and deaths globally. In 2020, it accounted for 2.5% of global deaths, and 6.5% of malaria cases in West Africa. Progress in malaria prevention and control has stagnated in recent years, with the estimated number of cases increasing by 10.4% between 2017 and 2020 [2]. Pyrethroid resistance has spread rapidly, and levels of resistance intensity are some of the highest in West Africa [16, 17].

The proposed study area is characterised by much higher malaria transmission intensity and more intense pyrethroid resistance in local vectors compared to previous cRCTs done in East and West Africa. For example, the entomological inoculation rate (EIR) ranged between 0.35 and 2.20 per night in several districts in central Côte d’Ivoire [18] compared to an EIR of 0.28 in Benin [19] and 0.15 in Tanzania [20]. It is, therefore, vital to demonstrate that these next-generation LLINs, which are becoming the standard of care in SSA, are superior to standard py-LLIN in the most extreme resistance areas, as this is likely where alternative interventions will be most needed to sustain gains in malaria control. This trial will generate the first epidemiological evidence on the efficacy of PBO-py LLINs compared to py-LLINs in West Africa. This protocol is reported in accordance with the Standard Protocol Items: Recommendations for Interventional Trials (SPIRIT) 2013 Statement [21].

### Objectives {7}

The main objective of this study is to use a cRCT to assess the efficacy of two types of dual AI LLIN for control of malaria compared to standard pyrethroid-only LLINs in Côte d’Ivoire where the main malaria vectors are highly resistant to pyrethroids.

#### Primary objective

Evaluate the efficacy of two dual AI LLINs compared to standard py-LLINs during 1 year on malaria case incidence in children aged 6 months to 10 years.

#### Secondary objectives

- To assess the efficacy of each of the two dual AI LLINs compared to a standard py-LLINs on: malaria infection prevalence (in all age groups at 6 and 12 months post net distribution) and vector density and EIR (as a proxy for malaria transmission for one year)
- To assess if dual AI LLINs have a safety profile similar to standard py-LLINs in the population of the trial study area

#### Tertiary objectives

- To understand the equity of LLIN coverage, usage, and benefits in the trial
- To monitor changes in insecticide resistance intensity and selection for resistance mechanisms over time and between arms
- To assess the impact of new types of LLIN on vector species composition

### Trial design {8}

The proposed trial will be a three-arm, superiority, triple-blinded cluster randomised trial, including 33 villages (11 in each arm), with an allocation ratio of 1:1:1. The village is the unit of randomisation (i.e. cluster). Each dual AI LLIN will be compared to standard LLIN. The 3 arms include (i) standard LLIN: MAGNet® LN [control/reference arm], (ii) mixture PBO-py LLIN: VEERALIN LN® [intervention 1], and (iii) mixture CFP-py LLIN: Interceptor® G2 LN [intervention 2].

## Methods: participants, interventions, and outcomes

### Study setting {9}

**Fig. 1:**
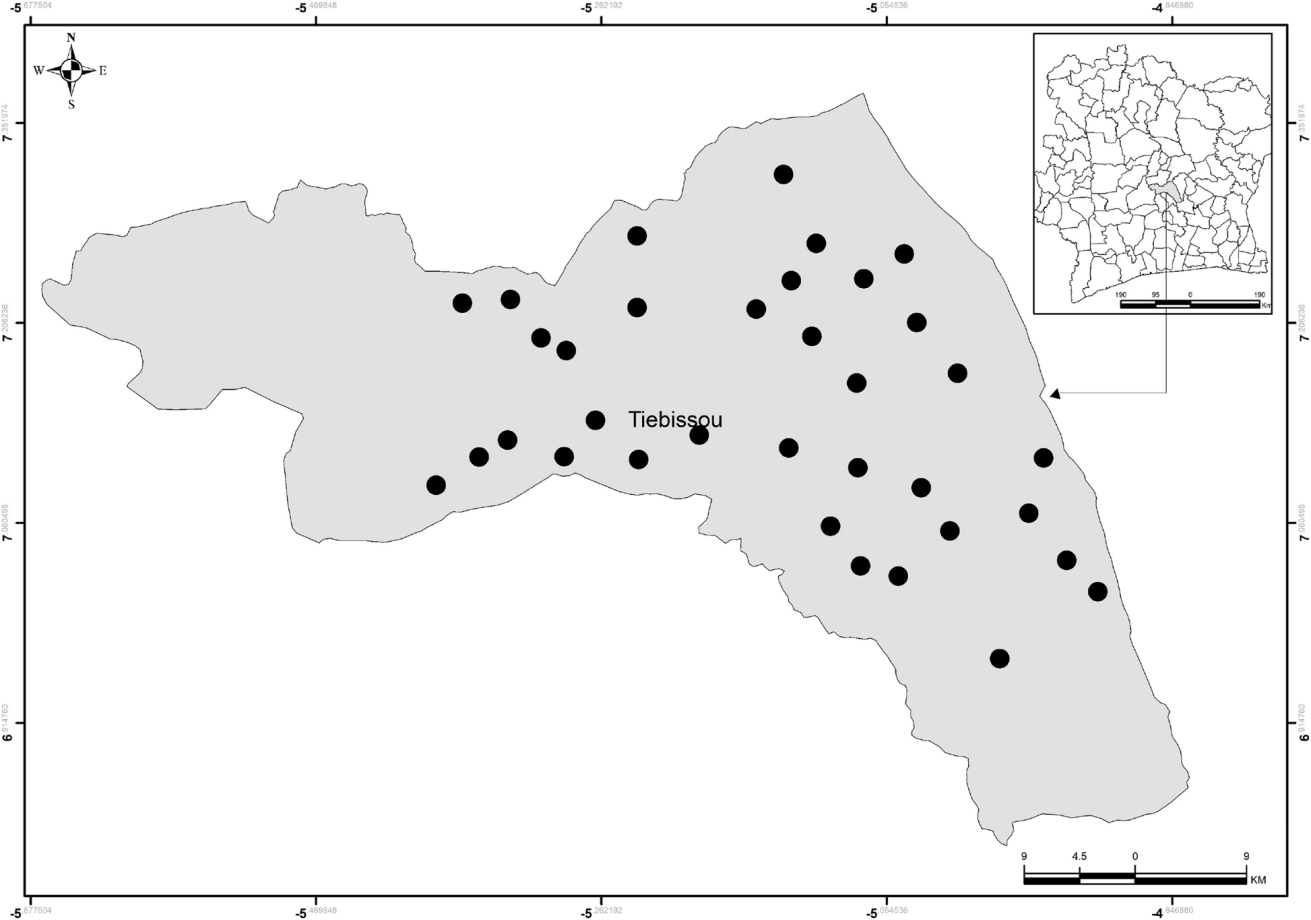
Map of Tiebissou department, central Côte d’Ivoire. Source of map: own from the study investigators (CS, ED)

The study will take place in the department of Tiebissou (Gbeke region, Lacs district), Southern Bouake city, central Côte d’Ivoire (Fig. 1). Tiebissou is about 40 km north of Yamoussoukro (political capital of Côte d’Ivoire) and around 60 km away from Bouake (second largest city in Côte d’Ivoire where Institut Pierre Richet (IPR) is based). The department of Tiebissou has 110 villages, with a population of about 116,321 in 2021, spread over 2410km^2^ [22].

There is one main malaria season from May to November. The Lacs district is characterised by intense indoor malaria transmission with a prevalence of malaria reaching 51.3% in children under 5 years old, according to the DHS conducted in 2021 [15]. Net usage for the same period and age group in the Lacs district was 55.3%.

Recent data in a neighboring district showed extremely high resistance intensity (> 1500-fold) to the pyrethroid deltamethrin in *An. gambiae* s.s. and *An. coluzzii*, and < 30% mortality after exposure to py-LLIN. The 1014F kdr mutation was almost fixed (≥ 90%). The carbamate and organophosphate resistance‐associated Ace-1 G119S mutation was also detected at moderate frequencies (22–43%). Transcriptomic analysis identified overexpression of P450 genes (i.e. Cyp6P3 expression up to 33-fold) [17, 23]. Data collected in 2021 by VectorLink demonstrated susceptibility of local vectors to chlorfenapyr in the Gbeke region [24].

### Eligibility criteria {10}

Thirty-three clusters will be included in this study based on the following criteria: being within a 2-h drive from Tiebissou town and at least 2 km away from other included clusters (in order to avoid cross-contamination), an estimated malaria prevalence of over 50% during baseline survey, and the acceptance of the hamlet leader and population.

The cohort will enroll randomly selected children (aged 6 months to 9 years old) who are resident in study villages and whose parents/caregivers have given written informed consent for their child to be included in the study. Children who are expected to be non-resident over the period of study will be excluded.

During cross-sectional malaria prevalence surveys, a maximum of 2 randomly selected household members who have resided in the village over the preceding 3 months, and who provide written informed consent for adults and parental/caregiver consent (and assent for children above 10) for children will be included. Vacant or unfound dwellings, severely ill inhabitants, and the absence of an adult capable of consenting will be reasons for exclusion.

### Who will take informed consent? {26a}

Prior to any project activities, village and hamlet leaders, as well as local health staff, will be invited to sensitisation sessions conducted by district health officers. Written informed consent will be sought before starting data collection from the local leader. Meetings will take place in each study cluster to ensure the community is informed as to the aims of the trial and the importance of utilising the nets that are distributed as part of the trial. Community health workers within each cluster will be fully informed as to the aims of the trial and will be on hand to answer day-to-day questions regarding the study.

For all activities (epidemiological and entomological data collection), written informed consent will be obtained from an adult guardian in the household or be given by the participant themselves if over 18 years. The consent form will be written in French and indicate the purpose of the study, the procedures, risks and benefits, that participation is completely voluntary, and that they may withdraw at any time. Study personnel will be trained in the oral translation of the information sheets and consent forms into the local dialects for participants who do not understand French. Participants will be asked to sign the consent in duplicate, one will be kept by the project, and they will remain with the other. If the person consenting is unable to read or write, their fingerprint will be taken, and a witness to the informed consent procedures signature will be requested to sign. For the active follow-up cohort, consent will be sought once at the enrolment visit of each cohort and will be performed by study nurses. For the cross-sectional surveys, which includes all ages, adults over 18 years of age will be asked to give informed consent for themselves and any children under 18 years, children older than 10 years enrolled for the prevalence cross-sectional survey would also be asked to assent. If consent is withdrawn, children will be able to leave the study at any point. Data up until the point of refusal will still be used if the participant agrees, if not, their data will be discarded. Where possible, they will be replaced in the cohort with a child from the same house.

Human Landing Catches (HLC) will be performed by non-pregnant volunteers above the age of 18 years. Informed consent will be obtained from these volunteers before being involved in the study. In order to prevent yellow fever, all volunteers will be vaccinated. They will be followed by the project clinical team and provided free treatment for malaria if necessary. The households where HLCs will take place will be randomly selected during each round of data collection and informed consent will also be obtained from the household head.

### Additional consent provisions for collection and use of participant data and biological specimens {26b}

De-identified blood samples collected for blood slides and filter paper blood spots will be stored for 2 years at Institut Pierre Richet and will be used for ancillary studies. Consent for the storage and use of biological specimens in this way and for the use of de-identified participant data for possible ancillary studies would be sought at enrolment. Study participants may voluntarily opt out on them without stopping participation in the trial.

## Interventions

### Explanation for the choice of comparators {6b}

#### Control: standard py-LLIN

MAGNet® LN (VKA Polymers Ltd, India) is a single pyrethroid-treated LLIN with alpha-cypermethrin (incorporated into filaments) at a target dose of 261 mg/m^2^ of polyethylene fabric (150 deniers). Standard py-LLINs are the current standard of care in Côte d’Ivoire alongside next-generation LLINs in some areas [25]. The safety profile of this net is well known. Some adverse effects associated with alpha-cypermethrin include skin irritation, eye tearing, sneezing, and headaches [12]. All adverse events will be closely monitored.

### Intervention description {11a}

#### Intervention 1: PBO-py LLIN

VEERALIN® LN is made of high-density polyethylene fibres, also manufactured by VKA Polymers Ltd, India, and incorporates alpha-cypermethrin at 216 mg/m^2^ and PBO at 79.2 mg/m^2^ of polyethylene fabric (130 deniers). VEERALIN ® LN has been shown to be more protective and potent at killing insecticide-resistant *An. gambiae* than py-LLIN and to be non-inferior to other PBO-py LLIN such as Olyset™ Plus and PermaNet® 3.0 in a direct comparison trial in experimental huts near Bouake [26]. PBO-py LLINs have never been tested in epidemiological trials in West Africa [9].

#### Intervention 2: CFP-Py LLIN

Interceptor® G2 LN, produced by BASF Corporation, is a mixture LLIN made of polyester coated with a wash-resistant formulation of 200 mg/m^2^ chlorfenapyr and 100 mg/m^2^ alpha-cypermethrin (100 deniers). CFP-py LLINs have been shown to be more effective and cost-effective than standard nets in Tanzania [12] and in Benin [13]. These nets are anticipated to become widely used across the sub-continent. Based on excellent results in experimental huts within the Bouake area, Interceptor® G2 nets are being distributed in targeted settings in Côte d’Ivoire [11].

The nets will be similar in colour, shape, and size. However, the Interceptor® G2 LN textile is different from the other two nets. We will therefore be monitoring closely net use to assess if the different textile will affect usage during the cohort visits and cross-sectional surveys. All three LLIN brands are pre-qualified by WHO PQT/VCP [5].

LLIN distribution will be done from a central location within each cluster. One LLIN will be given for every two people as recommended by the National Malaria Control Programme (NMCP) [25]. Census data will be used to calculate the number of LLIN needed for 100% coverage.

### Criteria for discontinuing or modifying allocated interventions {11b}

Not applicable because this is a cluster randomised trial with the intervention (bed nets) distributed in all clusters before the onset of participant enrolment. All adverse events such as skin reactions and cough will be carefully recorded by the study nurse, using adverse event and serious adverse event forms.

### Strategies to improve adherence to interventions {11c}

To maximise effective coverage of the nets for the purposes of this efficacy trial, a door-to-door hang up campaign will take place 1 or 2 weeks after the distribution. Information, education, and communication (IEC) activities will be conducted before, during, and after the LLIN distribution to increase usage in the study area, including instructions on how to wash the nets. Throughout the study, community health workers will encourage continuous net use in the study villages. During each cohort visit, study nurses will continuously encourage the use of LLINs.

The coverage achieved will be evaluated through each cohort visit as well as during cross-sectional surveys. Three indicators to evaluate net coverage will be used “proportion of households with at least one LLIN for every two people”, “proportion of households with enough LLINs to sleep under (access)”, and “proportion of residents reporting using a LLIN (study or not) last night”. We aim to reach 85% access following the distribution campaign and 75% usage [6, 13, 27, 28]**.**

### Relevant concomitant care permitted or prohibited during the trial {11d}

At enrolment into the cohort, all children will be cleared of malaria infection using artemisinin combination therapy (ACT), irrespective of malaria rapid diagnostic test (RDT) results. During cohort follow-up visits, malaria infection will be detected using the RDT and positive children will be treated according to national guidelines. Children will also be examined by study nurses during each follow-up visit for signs of other illnesses and will be treated if possible. There are no prohibited concomitant treatments.

### Provisions for post-trial care {30}

Post-trial, all clusters assigned to the control arm will receive Interceptor® G2 LN. It is expected to be the standard of care by the end of this trial [29]. There are no provisions for the compensation of study participants who suffer adverse events or serious adverse events because this study is considered to be minimal risk. Each type of net has undergone rigorous risk assessments following the WHO guidelines and is deemed unlikely to present risks to users at the insecticide concentrations applied to nets. The insecticides have a grade II or III WHO classification (unlikely to present a risk in normal use). Based on the WHO Pesticide Evaluation Scheme recommendation for Interceptor® G2 LN and PBO LLIN, sleeping under these nets does not pose undue risk to users when instructions are followed [30].

### Outcomes {12}

The different measurements which will be performed, and their frequencies are summarised in Table 1.

**Table 1:** Measurement type and frequency

Outcome	Measurement	Collection	Frequency
**Epidemiological outcomes**			
Malaria case incidence	Rapid diagnostic test taken when fever ≥ 37.5 °C and/or history of fever for the past 48 h	Active case detection: Cohort follow-up	Every 2 weeks during the transmission season/ every month during the dry season
Malaria infection prevalence	Rapid diagnostic test	Cross-sectional survey	Baseline, 6 and 12 months post net distribution
**Entomological outcomes**			
Indoor and outdoor Anopheles density	Human landing catch (HLC)	Entomology surveillance	6 houses per cluster every 2 months in all clusters for 12 months
Mosquito sporozoite rate	qPCR would serve to estimate the EIR [31]	Entomology surveillance	Subsample (30%) of the mosquito collected in HLC
Anopheles species identification	*An. gambiae, An.coluzzii* and *An. funestus* using Taq Man real-time PCR [32]	Entomology surveillance	Subsample of mosquitoes collected
Phenotypic resistance	WHO cylinder/CDC bottle assays [33]	Collection of larvae and/or adult *Anopheles* resting indoor	At baseline and at the end of the first year in 3 clusters per arm
Identification of molecular and metabolic insecticide resistance mechanisms	Screening for metabolic enzyme over-expression by RNA-seq Screening for resistance-associated mutations and copy-number variants by whole genome sequencing	Unfed 3-day-old adult *Anopheles* from larval collections phenotyped in bioassays	At baseline and at the end of the first year in 3 clusters per arm
Population-level genotypic resistance	Monitoring changes in key enzyme expression (identified by RNA-seq) using qRT-PCR Monitoring changes in frequencies of key mutations (identified by whole genome sequencing) using amplicon-seq	Unfed 3-day-old adult *Anopheles* from larval collections phenotyped in bioassays	At baseline and at the end of the first year in 3 clusters per arm
Serological responses to mosquito salivary peptides	Blood spots on filter paper will be utilised to measure the level of antibodies in residents blood as a proxy for exposure to mosquito bites	Cross-sectional survey	Baseline, 6 and 12 months post net distribution

*qRT-PCR* quantitative real-time polymerase chain reaction, *WHO* World Health Organization, *CDC* Center for Disease Control

The primary outcome measure will be incidence of malaria cases in children aged 6 months to 10 years over 1 year. A malaria case is defined as a fever above 37.5 °C or history of a fever in the last 48 h and a positive RDT. Malaria cases will be recorded at visits to cohort children, which will take place every 2 weeks during the malaria transmission season and every 4 weeks during the dry season.

Secondary outcomes include:

- Malaria infection prevalence (by RDT) in the study population of all ages at 6 and 12 months post net distribution.
- Vector density and EIR as a proxy for malaria transmission rate in the primary vector species.

Other outcomes are:

- Serological responses to mosquito salivary antigens as a proxy for exposure to bites: this will be measured by detecting antibodies to salivary peptide gSG6-P1 using enzyme-linked immunosorbent assay (ELISA)
- At baseline and 12 months post-net distribution: phenotypic resistance to alpha-cypermethrin, PBO + alpha-cypermethrin and chlorfenapyr will be assessed using WHO cylinder and CDC bottle bioassays. In addition, CDC resistance intensity tests with alpha-cypermethrin alone (5, 10, 15 × the diagnostic dose; DD) and following PBO pre-exposure will be performed.
- At baseline, we will identify mechanisms of insecticide resistance in the main vector populations using Illumina whole-genome sequencing and RNA-seq and develop trial site-specific panels of resistance markers for post-intervention monitoring. During the trial, selection for resistance will be monitored by measuring changes in levels of metabolic enzyme expression (using qRT-PCR) and frequencies of resistance-associated mutations and copy-number variants (using amplicon-sequencing), at 12 months post-intervention between study arms.
- Net quality: Bio-efficacy testing and chemical content of nets will be assessed after they arrive in Côte d’Ivoire and at 12 months post-intervention.

### Participant timeline {13}

**Fig. 2:**
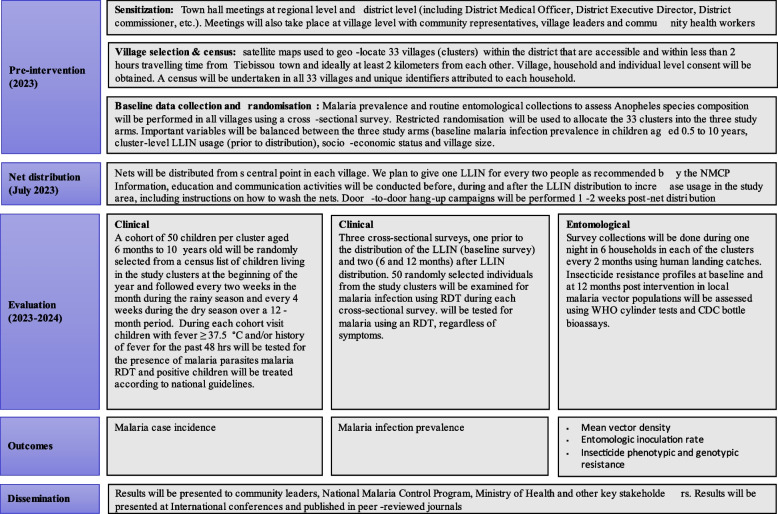
Trial design summary

A schedule of activities pre-intervention and post-interventions is shown in Fig. 2 (SPIRIT figure).

### Sample size {14}

Hayes and Bennett’s method was used in the sample size calculation for the primary outcome (malaria incidence in children aged between 6 months and 10 years) [34]. Based on data from a previous trial in a nearby area, the mean number of malaria episodes per child per year in the reference arm was assumed to be 1.2 with a between-cluster coefficient of variation of 0.29 [35]. Malaria case incidence in children under 10 years of age was reduced by 54% in Interceptor® G2trial arms in both Benin and Tanzania cRCTs at 12 months [12, 13] and by 47% in the PBO-py LLIN arm in Tanzania compared to py-LLINs in the first year [12]. In an area of higher resistance, intensity models have shown that impact of the PBO-py LLIN may be lower [36]. Hence, we have assumed that the interventions will reduce malaria incidence by 35% (i.e. to 0.78 cases). With a follow-up of 12 months, to detect this impact with 80% power would require follow-up of 45 children (plus 5 more children to account for potential losses to follow-up) in 11 clusters in each of our arms. No adjustment for multiplicity of testing has been included in the sample size estimations because the proposed designs and treatments test different research hypotheses [37, 38]. This results in a total of 33 clusters required for the trial and a cohort of 1650 children.

Sample size considerations take into account the clustered design [34] using prevalence of infection in each study arm as the secondary outcome, based on a superiority design. It was assumed that malaria prevalence in the reference arm has a mean of 50% with a coefficient of variation of 0.29 [35]. With 50 individuals per cluster and 11 clusters per arm, the study will have 80% power to detect a relative 35% lower prevalence (prevalence ratio 0.65) between each intervention arm compared to standard LLIN.

### Recruitment {15}

Each cluster will comprise one village with a minimum of 100 households and with a minimum of 100 children under the age of 10 identified during the census to increase the chances of recruiting sufficient numbers of study participants. A town hall meeting about the research project and activities will be conducted in all village clusters in the presence of health district leaders and at the regional level at the beginning of the project. To ensure support and that communities are well-informed on the trial, meetings will also take place at village level with community representatives. Village leaders and community health workers will be involved in each with the project team to insure adherence of all the population. Consent will be sought from hamlet leaders and household heads to ensure we achieve an adequate sample size. Research assistants, community health workers, and study nurses will be fully trained on the trial and always be available to answer any questions concerning the study.

## Assignment of interventions: allocation

### Sequence generation {16a}

Restricted randomisation will be used to allocate the 33 clusters into the three study arms. Important variables will be balanced between the three study arms (baseline malaria infection prevalence in children aged 0.5 to 10 years, cluster-level LLIN usage (prior to distribution), socio-economic status and village size). The randomisation method will allow us to limit the difference of these selected variables between the arms. At least 10,000 sequences will be generated and one will be randomly picked using a random number generator.

### Concealment mechanism {16b}

Nets will be identified using coloured loops and bales specific to the net type. Randomisation will be performed using the loops to identify the net types to ensure the statistician doing the randomisation is blinded to the allocation.

### Implementation {16c}

The cluster allocation sequence will be generated by the study statistician and the unblinded sequence held by an independent statistician.

## Assignment of interventions: Blinding

### Who will be blinded {17a}

Cluster inhabitants, trial participants (and their care providers where appropriate), data collectors (field staff who will collect mosquito and blood samples), and the study’s data analyst will be blinded. Cluster assignment will be listed by the color of the bale for each of the nets to ensure field implementers are also blinded to the net types.

### Procedure for unblinding if needed {17b}

The data will be shared with the DSMC who can request unblinding from the unblinded statistician if they have any concerns.

## Data collection and management

### Plans for assessment and collection of outcomes {18a}

#### Census

A pretested, short questionnaire including name of the head of the household, number of people living in the house, and the number of children in each age group (0–5 years, 5 to 10 years, 10 to 15 years, over 15 years)) will be administered. The census will be used to estimate the number of LLINs required to be distributed in each house and to randomly select children for the cohort and participants in the cross-sectional surveys. Every building of each village will be mapped using the Global Positioning System (GPS) function on a smartphone.

#### Cohort monitoring

All the children in the cohorts will be cleared of infection at the beginning of the enrollment by giving an artemisinin-based combination therapy. During enrollment visits, detailed questionnaires will be administrated collecting information about socioeconomical status, housing type, and results of the clinical examination. In the subsequent visits, a questionnaire will also be administered to inquire about LLIN usage the night before the visit, any adverse events encountered and travel history since the last visit. Cohort visits will take place every 2 weeks during the malaria transmission season and every 4 weeks during the dry season. A child will be considered ‘lost to follow-up’ if they miss at least 4 visits in a row (i.e. 8 weeks of follow-up time). Any child diagnosed with malaria will be provided with treatment following national guidelines.

In order to estimate malaria transmission in the broader study population, passive case detection will take place in health clinics located within catchment areas of the study clusters. Registration documents already in place in the health clinics include collecting data regarding the village of residence, age, pregnancy status, and gender- and whether they received a malaria test and the result of the test. This data will be collected from health clinics by study team members every 3 months and entered into an Open Data Kit (ODK) form.

#### Cross-sectional surveys

We will conduct three cross-sectional surveys, one prior to the distribution of the LLIN (baseline survey) and two (6 and 12 months) after LLIN distribution. Fifty randomly selected individuals from each study cluster will be examined for malaria infection during each cross-sectional survey. The survey will consist of (1) a household survey and (2) a clinical survey. During the household survey, a questionnaire will be administered to obtain information on possible risk factors for malaria and the randomly sampled person will be tested for malaria using an RDT, regardless of symptoms. A drop of blood will also be used to make microscopy slides to be assessed by study nurses and to be blotted on to filter paper, to detect antibodies to mosquito salivary peptides.

#### Entomology activities 

Entomological data collection will include collecting malaria vectors in each cluster every 2 months via HLCs. Data will be generated to calculate mean vector density and EIR for each arm in the trial.

The cross-sectional entomological monitoring will be done in all the clusters. Each cluster will be visited once every 2 months. Survey collections will take place during one night in 6 households in each of the clusters. In each survey, the six households will be selected at random from a census list of households in each study cluster at each timepoint and a description of the household will be recorded on a tablet. The questionnaire will include information on the number of inhabitants, type of house (wall, roof, number of rooms, number of sleeping places, etc.), and presence of animals. Collections will be done by HLC indoors and outdoors. We will use four volunteers for each household collection night, two indoors and two outdoors, with takeover in midway of the collection process during the night. Mosquito collections will run from 18:00 to 08:00 the next morning. The person doing the indoor catches will sit in the living room of the house and the one outdoors will sit on the veranda. The HLCs will be carried out using an aspirator. The volunteers will be lay-persons selected from the study villages and will be fully trained in the HLC activities.

Mosquitoes caught will be counted and identified using a species key based on morphological traits [39, 40]. A subset of the captured female *Anopheles spp* vectors belonging to the *An. gambiae* complex, *An. funestus*, and *An. nili* groups will be stored in individual capsules and kept for sibling species by PCR [32, 41, 42] and sporozoite analysis by PCR [31]. A subsample of *Anopheles* from each cluster will be stored in RNA-later® to compare metabolic enzyme profiles between arm and over time.

To determine insecticide resistance profiles at baseline and at 12 months post intervention, *Anopheles* larvae will be collected from 3 clusters per trial arm c, reared to adult and exposed to different insecticides. WHO cylinder tests and CDC bottle bioassays will be performed using diagnostic concentrations (DCs) of alpha-cypermethrin, PBO + alpha-cypermethrin, and chlorfenapyr. In addition, CDC resistance intensity bioassays for alpha-cypermethrin and PBO + alpha-cypermethrin (5, 10 or higher x DD) will be performed. Whole transcriptomic characterisation (RNA-seq) will be used to identify highly over-expressed genes per phenotype and will form the basis of site-specific qRT-PCR panels for post-intervention (and wider population-level) resistance monitoring. Illumina whole genome sequencing will be used to identify genetic variants (single-nucleotide polymorphisms and/or copy-number variants), associated per phenotype and will form the basis of site-specific amplicon-seq panels for post-intervention (and wider population-level) resistance monitoring.

#### Quality assurance of LLINs

At the beginning and end of trial, we will conduct cone bioassays using a laboratory colony of susceptible *Anopheles gambiae* Kisumu strain to evaluate quality of the LLINs when they are new. Five nets per types will be randomly selected. From each of the LLINs selected, one net piece measuring 30 cm × 30 cm will be cut from each side. Fifty *An. gambiae* (Kisumu strain) will be tested per selected net (total of 250 females per treatment arm). A separate piece cut from the selected net will be sent to Gembloux (Belgium) for chemical testing.

### Plans to promote participant retention and complete follow-up {18b}

After enrolment into the cohort, every reasonable effort to complete follow-up will be made by the study staff within each cluster. The staff will make home visits and use alternate contact information to determine the whereabouts of the child. The projected loss to follow-up rate is 10% and the sample size has been inflated to account for this. Site study staff will develop and implement local standard operating procedures to achieve the projected follow-up rate.

### Data management {19}

Household data, clinical measurements in cohort incidence study, and entomological data collected during the cross-sectional surveys will be captured on electronic forms using smartphones installed with ODK Collect. The data will be stored on a secure server located at LSHTM and all data management and manipulation will be done using Stata (Stata Corp). Laboratory data output will be available directly from the analyser (e.g. qPCR data) and imported into a database. Data extractions will be converted into Stata format for querying and analysis. It will be possible to share de-identified data in several widely used formats.

#### Data quality and control

Paper case report forms (CRFs) will have numbered and coded items to ensure straightforward and accurate data entry and processing, and drafts will be reviewed by the study team before finalisation. Standard Operating Procedures (SOPs) for data collection will be developed and field staff will be appropriately trained to ensure rigorous data collection. This will include quality control (QC) of their own performance by checking for missing data or implausible responses. Furthermore, more QC checks will be performed by a supervisor to check for data completeness and internal consistency of responses within a few hours of data collection. Corrections, when appropriate, will be done before the CRFs are submitted for data entry. Electronic CRFs will have built in checks for missing data, implausible responses, and internal consistency; data collected using electronic CRFs will include the device serial number and date/time stamp and the device will be password protected. All quantitative data collected on paper CRFs will be double-entered into a database independently by two data clerks. The database will maintain an audit trail with time-date stamps of data entry and all changes that are made to the data. Records of the username of the person who did the data entry and/or changed the record will be kept. A series of QC checks will be conducted during the initial review of the completed CRFs before data entry, during data entry, and during data validation. The checks which will be performed on all data will be specified in a detailed document (data checks document), which will be developed by the Data Manager and approved by the study team. This will include checks for range, values, and missing data. During the study, the data manager will prepare a regular QC report to document the current status of data entry and any corrective actions needed; this will be communicated to the field supervisors for resolution. In addition, before the database is locked, the data manager and team will perform a QC check of the database against the CRFs. This check will be based on a risk assessment and may, for example, include a 100% check of key variables, and a 10% check of the remainder of the database. After QC, and with agreement of the study team, the data manager will lock the database, withdrawing all write/edit permissions so that no further changes can be made.

#### Data security 

Every effort will be made to ensure data security, particularly relating to sensitive participant information. All data will be uploaded onto a secure server on the LSHTM cloud. All data will be stored encrypted and will be accessible only by password and encryption keys held by the data manager. In the study database, we will not store any information that could be used to identify individual study participants. We will use anonymised study numbers as our unique participant identifier.

The main risk to the confidentiality and security of information are names and housing coordinates acquired to link malaria prevalence results to geographical areas. This risk is managed by giving a unique identifier number to each participant and household. This confidential data is only managed by the project manager and will be kept separate from other databases in the short to medium term. It will then be stored securely and not on a shared drive so that anonymity is not lost in the long term.

#### Data storage 

Upon completion of the study, electronic files will be stored on a server and also copied to encrypted USB and stored offsite in a safebox. CRFs will be stored in the secure archive, which is equipped with locked cabinets for long-term storage of CRFs and documents. All paper source records will be retained for a minimum of 10 years from the point of publication of data on the primary outcome. Electronic data will be stored for a minimum of 10 years following study completion, with regular checks to make sure that the data are still readable.

### Confidentiality {27}

All procedures for data collection, management, storage, and manipulation will follow SOPs. To ensure confidentiality is maintained, paper CRFs with participants’ names will be stored in a locked cabinet and only accessible by authorised staff. This risk is also managed by giving a unique identifier number to each participant. All analyses will be done using the unique ID.

### Plans for collection, laboratory evaluation, and storage of biological specimens for genetic or molecular analysis in this trial/future use {33}

De-identified blood samples collected for filter paper blood spots and blood slides will be stored for 2 years at IPR and will be used for the current trial and potential future studies. Samples will be stored with a dessicant in a fridge at 4 to 8 °C). Samples will be identified using a QR code.

## Statistical methods

### Statistical methods for primary and secondary outcomes {20a}

Descriptive statistics (mean and standard deviation or median and interquartile range for continuous variables, and frequency and proportion for categorical variables) will be used to compare characteristics of participants between the study arms. All primary analyses will be conducted by intention to treat (ITT).

#### Primary outcome

The main analysis will be done on malaria case incidence collected over 12 months. Incidence of malaria cases in each dual AI LLIN treatment arm ( VEERALIN®LN and Interceptor® G2 LN) will be compared to the incidence of malaria cases in the reference arm (MAGNet® LN) to assess whether the new dual AI LLINs are superior to the reference LLINs. Children will be followed up for 12 months. Following any treatment for malaria, a child will not be considered at risk for 2 weeks and this period will be censored.

All analyses will be based on comparisons of incidence rates between clusters randomised to the three arms. Due to the relatively small number of clusters per arm (*n* = 11), analyses will take place at the cluster level. A summary measure will be obtained for each cluster and the arms will be compared using a two-sample *t*-test [34]. If the cluster-specific observations have a skewed distribution, they will be logarithmically transformed prior to the *t*-test.

#### Secondary outcomes

Prevalence of malaria infection in the study clusters will be estimated at baseline and 6 and 12 months. Prevalence will be measured in all age groups in the study clusters. As for the incidence outcome, due to the relatively small number of clusters, analyses will primarily take place at the cluster level, with cluster-level proportions compared using a *t*-test. Depending on the distribution of proportions at the cluster level, a logarithmic transformation may be used before the *t*-test. The primary comparison will be 12 months post net distribution.

The level of antibodies to mosquito salivary peptides will be measured in samples taken at baseline and 6 and 12 months after net distribution in the same population randomly selected to take part in the infection prevalence survey. Serological outcomes will be expressed in proportion positives with the threshold for positivity defined using a mixture model.

EIR will be estimated as *Anopheles* density collected in the HLCs per house per night multiplied by the proportion of sporozoite-infected vectors*.* Cluster-level results will be compared using a *t*-test, using a logarithmic transformation if necessary.

Adverse events (AEs) and serious adverse events (SAEs), their severity and relation to the intervention will be examined and tabulated by study arms.

For whole transcriptomic sequencing, total RNA will be isolated from pools of 5–10 *An. gambiae* s.s., *An. coluzzii*, or *An. funestus* s.s., per phenotypic group (i.e. “resistant”—survived exposure to five or ten times the discriminating insecticide concentration; or “unexposed”—mosquitoes from the same field population, which were not exposed to insecticide in control bioassays) and used to prepare RNA-seq libraries for next-generation sequencing, following ribosomal RNA depletion. To obtain sufficient sequencing coverage and depth, our previous RNA-seq experiments have typically comprised nine libraries (two wild populations in technical triplicate and one susceptible colony comparator, also in triplicate—either *An. gambiae* s.s. Kisumu, *An. coluzzii* N’Gousso, or *An. funestus* s.s. FANG), sequenced at 2 × 125 bp paired-end reads on an Illumina Hi-Seq platform [18]. Following sequencing read alignment to annotated reference transcriptome assemblies, available from VectorBase [43] differentially expressed genes (DEGs) will be identified using the standardised DESeq2 pipeline [44]. To account for induction of gene expression during insecticide bioassays, pairwise comparisons of DEGs between “unexposed” field mosquitoes and “susceptible” colony mosquitoes will allow for the identification of significantly over- or under-expressed RNA transcripts, which are associated with constitutive resistance, while comparisons between “resistant” field mosquitoes and “unexposed” field mosquitoes will detect changes in gene expression in response to chemical exposure. Candidate genes, implicated in particular resistance profiles, will be validated by real-time quantitative PCR (RT-qPCR) and developed into multiplex TaqMan RT-qPCR assays to use for surveillance of insecticide resistance selection and changes in relative intervention performance in the cRCT [45].

For whole genome sequencing, total DNA will be isolated from individual *An. gambiae* s.s., *An. coluzzii*, or *An. funestus* s.s., per phenotypic group (i.e. “susceptible”—died following exposure to the discriminating insecticide concentration; or “resistant”—survived exposure to five or ten times the discriminating insecticide concentration) and used to prepare multiplex libraries for sequencing at 2 × 150 bp paired-end reads on an Illumina Hi-Seq platform. Sequence reads will be aligned to the appropriate species reference genome (*An. gambiae* s.s.: AgamP3; *An. coluzzii*: AcolN1.1; or *An. funestus* s.s. AfunF3) from VectorBase [43], using bwa-0.7.9-r783, sorted using samtools 0.1.17, and potential genetic variants (including single-nucleotide polymorphisms and insertion/deletions) will be identified using the Genome Analysis Toolkit (GATK) v4.2.2.0. and copy-number variants (CNVs) and structural re-arrangements will be screened for using Hidden Markov Models (HMMs) [46]. Genetic variants will be developed into novel, custom insecticide- or intervention-specific amplicon-sequencing panels for surveillance in the cRCT [47].

### Interim analyses {21b}

There will be no interim analyses. The Data Safety Monitoring Committee (DSMB) will have access to the data to ensure that the study is appropriately powered and that no adverse events are occurring due to the intervention.

### Methods for additional analyses (e.g. subgroup analyses) {20b}

The impact of covariates such as baseline prevalence age of child, gender, socio-economic status, distance to health facility, etc. on malaria incidence will be analysed, where regression is carried out on the individual-level data (ignoring the clusters) and then comparing the residuals for each cluster based on the observed outcome, compared to the predicted outcome, in the absence of an intervention effect. Comparison of the residuals using a *t*-test will provide a measure of intervention effect, adjusted for covariates.

For malaria infection prevalence data, subgroup analysis will include investigating individual level, household level, distance to health facility, and cluster-level characteristics, such as age group, socio-economic status, household construction, vector species composition, cluster net coverage, and insecticide resistance intensity. These analyses will be performed on the residuals of the cluster-level results, as documented for the incidence analyses. Secondary analyses with EIR data will include adjusting for timepoint and comparing residuals of the cluster-level summaries.

Where possible, geospatial models of vector species distribution and insecticide resistance across the study area will be developed using machine learning algorithms incorporating entomological field indices linked to the point locations of vector sampling alongside earth observation data on relevant covariates (e.g. climate, vegetation indices, elevation, land cover/land use, population density).

### Methods in analysis to handle protocol non-adherence and any statistical methods to handle missing data {20c}

We do not expect that there will be significant cross-over (by having a minimum distance of 2 km between study clusters, thereby creating buffers between study clusters) or loss to follow-up. Hence, further analyses to account for these factors should be very similar to the ITT analyses. Separate analyses will be done looking at regular net users compared to those who report irregular use, that is, < 75% reported usage the previous night over the entire follow-up duration. During cross-sectional surveys, separate analyses will be performed to compare people reporting the use of the correct net the previous night to those who did not.

Reasons for withdrawal from each treatment arm will be reported and the reasons compared qualitatively. Missing data will be described in each arm and possible biases due to missing data discussed. Data analyses will be performed on complete cases. The possible effect of missing data on study results will be evaluated in sensitivity analysis.

### Plans to give access to the full protocol, participant-level data, and statistical code {31c}

The full protocol, future participant-level dataset, and statistical code will be available on reasonable request.

## Oversight and monitoring

### Composition of the coordinating centre and trial steering committee {5d}

A Trial Steering Committee (TSC) of three Africa-based experts in Entomology and Epidemiology with considerable experience of vector control trials will be established to provide oversight for the study. The TSC members will be independent of the trial and its institutions and have the necessary expertise to monitor study progress and participant safety. They will meet remotely every 6 months to get an update on the trial. The coordinating centre will be run by an experienced management team, a trial manager, data manager, and field teams.

### Composition of the data monitoring committee, its role and reporting structure {21a}

A DSMC will be established to provide oversight for the study. This committee will be made up of three expert Statisticians and Epidemiologists, with experience in analysing trial data. DSMC members will be independent of the trial and its institutions and have the necessary expertise to monitor study progress and participant safety. The DSMC will be responsible for monitoring the progress of the trial, adherence to the protocol, the safety data, and the critical efficacy endpoints. Members will meet remotely or in-person every 6 months.

### Adverse event reporting and harms {22}

Although the risk to study participants is considered minimal from any of the study LLINs, we will still document their safety. Any adverse events which have been reported previously to be associated with exposure to insecticides such as skin rashes, skin burning, skin itching, skin paraesthesia, eye tearing, watering eyes, runny nose, sneezing, mucosal irritation, headache dizziness, and their severity will be monitored in the study cohort and study participants selected during the cross-sectional survey. We will also record other AEs and SAEs will be collected during follow-up of the cohort children by non-directive questions during the follow-up visits and through volunteer reporting in the study population. Study staff will record any AEs in the AE report form and immediately report to the study clinician. Excessive clustering of SAEs will be reported to DSMC, TSC, and IRB. Study personnel will be trained to recognise and report AES.

The trial will follow standard definition for AEs and SAEs [48]:

- Adverse events: any untoward medical occurrence in a patient or clinical investigation subject administered a pharmaceutical product and which does not necessarily have to have a causal relationship with this treatment.
- A serious adverse event (experience) or reaction is any untoward medical occurrence that at any dose: (1) results in death, (2) is life-threatening, (3) requires inpatient hospitalisation or prolongation of existing hospitalisation, (4) results in persistent or significant disability/incapacity, or (5) is a congenital anomaly/birth defect.

### Frequency and plans for auditing trial conduct {23}

The trial management team will be responsible for trial conduct and will report to the TSC at regular timepoints throughout the trial. The TSC are independent from the study sponsor and investigators.

### Plans for communicating important protocol amendments to relevant parties (e.g. trial participants, ethical committees) {25}

Any amendments to the study protocol and informed consent forms will be submitted for approval to both IRBs. The funder and sponsor will be notified of any changes to the protocol, as well as the TSC and DSMC. A copy of the amended protocol will be added to the Trial Master File. Protocol deviations and/or violations will be documented using protocol deviation notification forms.

#### Patient public involvement

There was no patient or public involvement in the design of this protocol.

### Dissemination plans {31a}

Quarterly progress reports will be shared with district and NMCP officials to disseminate project findings and improve malaria knowledge, local strategy, and LLIN usage. An end of project workshop with MOH, NMCP, implementing partner, and funders will be organised to share study findings and translate into future vector control strategy. Mid- and end of project meetings will be held to share progress and results with village and community leaders to help disseminate findings to the community. Keynote presentations by project researchers at international scientific conferences will be done to present study findings to the international malaria control community.

## Discussion

There is a paucity of data on the efficacy of pyrethroid-PBO LLINs on malaria case incidence and malaria infection prevalence, especially in West Africa, where vectors tend to exhibit high levels of insecticide resistance and mechanisms of resistance may differ from those in East African vector populations. The efficacy of chlorfenapyr-py LLINs compared to standard py-LLIN has been demonstrated in cRCTs in Tanzania [12] and in Benin [13] over a 2-year follow-up period. This led to the WHO revising its recommendations earlier this year. The WHO now strongly recommends the use of chlorfenapyr-py LLINs instead of standard py-only LLIN in areas with insecticide-resistant malaria vectors [14]. However, there is still a lack of evidence on the efficacy of py-PBO LLINs compared to standard py-only LLIN in West Africa on epidemiological and entomological outcomes. In previous trials, PBO-pyrethroid nets seemed to have marginal superiority over pyrethroid-only nets at 24 months post net distribution [7, 12, 13].

To the best of our knowledge, this will be the first trial of py-PBO nets in West Africa. This will add to the existing body of literature by providing much needed data in this high-transmission setting. The results of this trial will be used by the WHO for informing malaria control policies in West Africa.

## Trial status

Protocol version 1.1 of December 08 2022. Participant recruitment has not begun. Participant recruitment is estimated to end June 30, 2024.

## Data Availability

The Principal Investigator (PI), Jackie Cook and Co-PI Raphael N’Guessan, will have full access to all the data in the study and will have final responsibility for the decision to submit for publication.

## Abbreviations

AE: Adverse event
AI: Active ingredient
CFP: Chlorfenapyr
CFP-py: Chlorfenapyr-pyrethroid
COV: Coefficient of variation
cRCT: Cluster randomised control trial
CRF: Case report form
DHS: Demographic health survey
DSMC: Data safety monitoring committee
EIR: Entomologic inoculation rate
ELISA: Enzyme-linked immunosorbent assay
GIS: Geographical information systems
GPS: Global positioning system
ICC: Intra-cluster correlation coefficient
IEC: Information, education, communication
IG2: Interceptor G2
INSP: Institut National de Santé Publique
IPR: Institut Pierre Richet
IRB: Institutional review board
ITT: Intention to treat
HLC: Human landing catch
LLIN: Long-lasting insecticidal net
LSHTM: London School of Hygiene and Tropical Medicine
NMCP: National Malaria Control Program
ODK: Open Data Kit
PBO: Piperonyl butoxide
PBO-py: Piperonyl butoxide- pyrethroid
PCR: Polymerase chain reaction
Py: Pyrethroid
RDT: Rapid diagnostic test
RR: Rate ratio
SAE: Serious adverse event
SOP: Standard operating procedure
SSA: Sub-Saharan Africa
TSC: Trial steering committee
WHO: World Health Organization
